# From ranking to decision: TASC-VS for calibration-aware top-k virtual screening

**DOI:** 10.1371/journal.pone.0356482

**Published:** 2026-08-19

**Authors:** Lisha Zou, Shiyu Tian, Zhipiao Tang, Keyi Zou

**Affiliations:** Hunan University of Information Technology, Changsha, Hunan, China; AlloTec Bio, UNITED STATES OF AMERICA

## Abstract

Virtual screening is most useful when it improves top-*k* prioritization under limited experimental budgets. We evaluated the full TASC-VS package on a curated 15-target subset of the LIT-PCBA virtual-screening benchmark. Methodologically, TASC-VS combines test-time adaptation (TTA), calibration, and uncertainty-aware ranking to support fixed-budget decisions. Coverage analysis showed that the subset is not full LIT-PCBA: it contains 23,250 evaluated molecule-target records and 181 positives, with positives in seven targets. Empirically, TASC-VS full showed modest, directionally consistent benefits over the evaluated comparators. On the seven positive targets, normalized enrichment factor at 1% (NEF1) and Boltzmann-enhanced discrimination of receiver operating characteristic (BEDROC) were 0.052 and 0.062 for TASC-VS full, compared with 0.0057 and 0.0279 for RTMScore-on-Vina-poses. Across all 15 targets, precision and false-positive count among the top 20 candidates (P20 and FP20) were 0.0433 and 19.13 for TASC-VS full, compared with 0.0067 and 19.87 for RTMScore-on-Vina-poses. Calibration improved reliability metrics, uncertainty penalization produced a small top-20 benefit, and TTA changed reliability slightly without driving the main ranking gain. Paired P20 analysis versus RTMScore-on-Vina-poses gave a mean benefit of 0.0367 (95% bootstrap confidence interval (CI), 0.0067–0.0733; raw Wilcoxon *p* = 0.026), but multiplicity-corrected values were not significant (Holm *p* = 1.0; Benjamini–Hochberg (BH) *q* = 0.322). An external Directory of Useful Decoys, Enhanced (DUD-E) stress test favored Vina over TASC-VS full. Thus, TASC-VS full provides calibration-aware top-*k* decision support on the curated LIT-PCBA subset; broader deployment requires separate validation.

## Introduction

In early-stage drug discovery, virtual screening is widely used to prioritize potentially bioactive compounds from large chemical libraries [1,2]. Its true value lies not merely in improving overall scoring performance or global ranking correlation, but in determining whether the most promising candidates can be placed reliably at the top of the list when experimental validation resources are limited. In recent years, as screenable chemical space has continued to expand, structure-based screening workflows have become increasingly mature, and computational outputs have been used more directly to guide downstream experimental decisions, top-*k* prioritization has become one of the most practically relevant problems in virtual screening [3–6]. At the same time, this task faces a persistent challenge: although many methods are available, their performance is often markedly heterogeneous across targets, and commonly reported benchmark-level metrics do not always adequately capture the real value of screening under fixed-budget validation settings. A practical question is therefore whether a complete screening package provides a reproducible, calibration-aware top-*k* decision advantage within a clearly specified benchmark.

Existing studies on top-*k* prioritization in virtual screening can be broadly grouped into three major methodological lines. The first consists of classical docking-centered structure-based screening approaches, which typically offer mature workflows, relatively high computational efficiency, and suitability for rapid primary screening of large candidate libraries, and therefore have long served as the fundamental comparison framework in virtual screening research [7–14]. The second includes rescoring strategies and machine-learning-based scoring functions, whose central idea is to improve scoring and ranking performance by exploiting richer protein–ligand representations after initial docking or pose generation, thus representing a major transition from empirical scoring toward data-driven screening pipelines [15–19]. Representative examples include RTMScore, delta-learning scorers, TB-IECS, PIGNet2, vScreenML, recent GNINA-family advances, and generalization-oriented analyses of learned scoring functions [20–29]. The third comprises consensus or multi-stage combination strategies, which do not assume that any single program can dominate consistently across all targets, but instead seek to improve early enrichment and hit discovery by integrating multiple docking programs or scoring outputs; such strategies are particularly useful when the cross-target stability of a single method is limited [30–36]. This line also overlaps with ensemble-docking and multi-stage campaign designs reported in recent application studies [37–41]. Overall, prior studies have established a relatively complete methodological landscape spanning classical docking, machine-learning-based rescoring, and combinatorial screening pipelines. Classical docking studies often emphasize pose generation and throughput, learned rescoring studies usually prioritize scoring or ranking performance, and consensus studies focus on combined enrichment. Calibration-aware fixed-budget decisions for complete screening packages remain less examined.

Two issues motivate a decision-focused evaluation. First, virtual-screening performance is often heterogeneous across targets, so aggregate means alone may obscure target-level gains and losses. Second, pose prediction, scoring accuracy, and global ranking metrics do not directly measure decision quality when only a small number of compounds can be tested. A method that performs well on summary metrics is therefore not necessarily the most useful choice for a fixed-budget screening campaign [42]. We address this gap by linking benchmark-level performance, target-level comparisons, and fixed-budget decision value.

**Fig 1:**
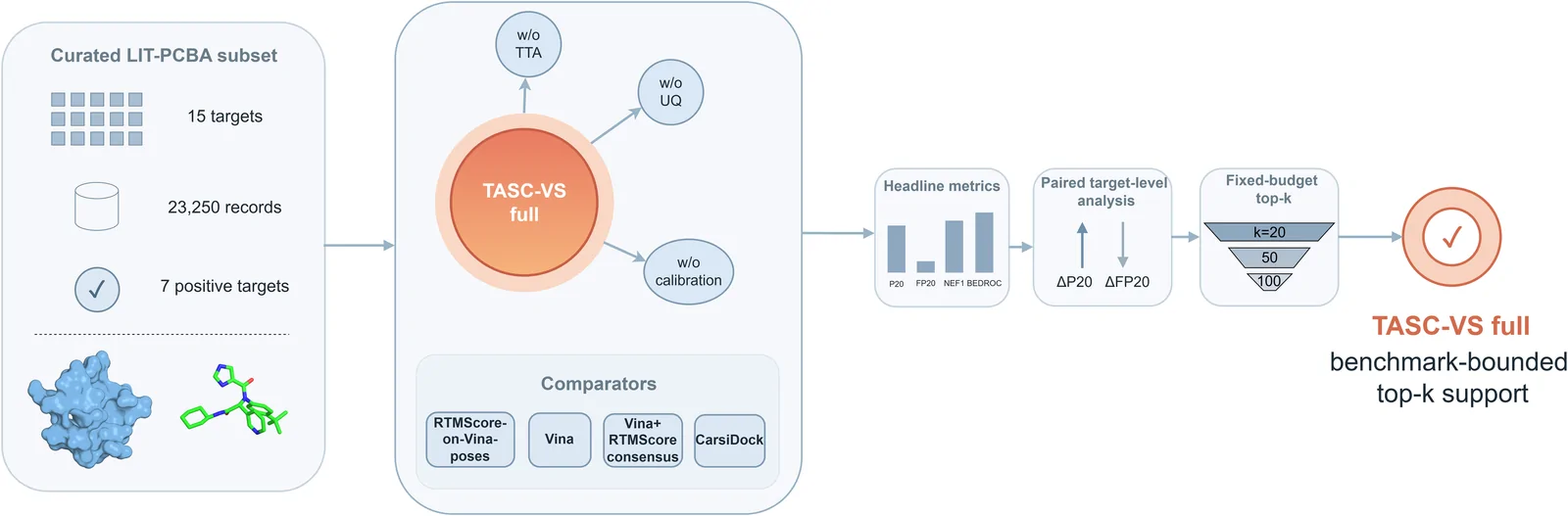
Overview of the frozen-benchmark comparison framework used in this study.

This study evaluates whether TASC-VS full provides a calibration-aware top-*k* decision advantage on a curated 15-target LIT-PCBA subset. We compared the full package with classical baselines and examined headline benchmark performance, paired target-level effects, and fixed-budget decision quality. The resulting conclusion is tied to the evaluated benchmark and comparator set. To provide a compact overview of the study design, Fig 1 summarizes the frozen-benchmark comparison framework used in this work. All methods were evaluated on the same frozen 15-target benchmark under an auditable comparison setup. The evaluation combined headline metrics with fixed-budget top-*k* analysis to assess calibration-aware decision value in virtual-screening prioritization.

The main contributions of this study are as follows:

- We formulate virtual-screening comparison as a calibration-aware top-*k* decision problem under fixed experimental budgets.
- We establish a multi-layer evaluation framework consisting of headline benchmark performance, paired target-level analysis, and fixed-budget decision quality, such that method comparison no longer relies solely on a single summary metric.
- We evaluate TASC-VS full against multiple baselines on the same curated benchmark subset using aggregate summaries, paired target-level effects, and fixed-budget endpoints.
- We shift the focus of evaluation from abstract metric superiority to decision value in realistic screening workflows, with particular emphasis on top-*k* prioritization quality under fixed-budget settings and its deployment relevance.
- We define the scope of the result through component analyses, target-composition sensitivity analyses, overlap filtering, modern-baseline feasibility checks, runtime estimates, and an external DUD-E stress test.

## Methodology

### Study design and benchmark data source

This study was designed as a deployment-oriented benchmark comparison. We asked whether TASC-VS full provides a calibration-aware advantage for fixed-budget top-*k* prioritization on a curated 15-target virtual-screening subset. The analysis focused on overall screening performance, decision quality under limited experimental budgets, and the scope of the observed benefit.

The evaluation used a curated subset of 15 LIT-PCBA targets [43]. LIT-PCBA is an unbiased virtual-screening benchmark designed for controlled method comparison, but the subset evaluated here is not the full benchmark. Inclusion required target metadata, fold assignment, receptor preparation, ligand labels, Vina poses, RTMScore outputs, and the input features required by TASC-VS. The resulting subset contains 23,250 evaluated molecule-target records and 181 positives, whereas the published LIT-PCBA benchmark contains 415,225 molecules and 7,844 actives across its full target collection. The present analysis therefore covers approximately 5.6% of published molecules and 2.3% of published actives. Positives are present in seven of the 15 evaluated targets. S1 Table reports target-level coverage. This restricted subset supports a controlled package comparison, while benchmark-construction and hidden-bias issues remain relevant across virtual-screening datasets [44–47].

The 15 evaluation targets were ALDH1A1, MTORC1, VDR, ESR1_ANTAGONIST, FEN1, OPRK1, GBA, MAPK1, PPARG, ADRB2, KAT2A, PKM2, ESR1_AGONIST, IDH1, and TP53. Target-specific total/positive counts were 58/4, 1475/3, 63/0, 3787/77, 62/0, 63/0, 61/0, 63/0, 3929/23, 5964/0, 59/0, 62/1, 4243/12, 62/0, and 3299/61, respectively. Because several targets contained no positives, we defined two analysis sets before interpretation: an all-target deployment set, used to reflect screening utility and false-positive burden across the full frozen target portfolio, and a positive-target subset restricted to targets containing at least one positive, used for enrichment and related metrics that require positive support.

The study used five fixed outer folds to ensure target-level reproducibility. Fold A contained ALDH1A1, MTORC1, and VDR; fold B contained FEN1, ESR1_ANTAGONIST, and OPRK1; fold C contained MAPK1, GBA, and PPARG; fold D contained KAT2A, PKM2, and ADRB2; and fold E contained TP53, IDH1, and ESR1_AGONIST. Validation rotation was fixed as A→B, B→C, C→D, D→E, and E→A, so that for each outer fold the current fold was used for testing, the next fold for validation, and the remaining nine targets for training. For folds A through E, the train/validation/test record counts were 17,742/3,912/1,596, 15,285/4,053/3,912, 13,112/6,085/4,053, 9,561/7,604/6,085, and 14,050/1,596/7,604, respectively. This structure provided fixed target-level isolation for training, calibration, and held-out evaluation.

### Compared methods and experimental workflow

An overview of the comparison framework is shown in Fig 1. The compared methods were the full TASC-VS package (TASC-VS full), TASC-VS without test-time adaptation (TASC-VS without TTA), TASC-VS without uncertainty quantification (TASC-VS without UQ), TASC-VS without calibration, RTMScore-on-Vina-poses, Vina, Vina+RTMScore-on-Vina-poses consensus, CarsiDock, and a VSFlow ligand-similarity baseline. Separate feasibility analyses examined GNINA-on-Vina-poses and DrugCLIP with reconstructed pockets. Molecule-level scores were generated under the same benchmark split whenever possible, compounds were ranked within each target, and the ranked outputs were summarized using headline metrics, paired target-level comparisons, fixed-budget endpoints, component ablations, sensitivity analyses, and an external stress test.

TASC-VS is a fold-wise learned rescoring model with a fixed 175-dimensional input representation. The input vector contains three scalar upstream scores or energies (Vina affinity, RTMScore score, and selected conformer energy), 32 RDKit molecular descriptors, 12 pose-interaction descriptors derived from the docking pose, and a 128-dimensional hidden representation extracted from the official pretrained RTMScore model. The RTMScore score and embedding therefore use the pretrained RTMScore model; the local TASC layer is trained only on the LIT-PCBA training targets defined by each outer-fold split.

The base TASC model is a multilayer perceptron that maps the 175-dimensional feature vector to one activity logit. The architecture is Linear(175,512)–GELU–LayerNorm–Dropout(0.2)–Linear(512,256)–GELU–LayerNorm–Dropout(0.2)–Linear(256,64)–GELU–Linear(64,1), where GELU denotes a Gaussian error linear unit and LayerNorm denotes layer normalization. For each outer fold, five independent checkpoints were trained with seeds 20260326, 20260327, 20260328, 20260329, and 20260330. Training used AdamW with learning rate 0.0002, weight decay 0.0001, batch size 1024, maximum 50 epochs, and early stopping patience of 8 epochs. To address class imbalance, mini-batches used a 1:20 positive-to-negative quota when both classes were available. The training loss combined 0.7 times focal binary classification loss with α=0.75 and γ=2.0 and 0.3 times pairwise ranking loss with margin 0.2. Here, α weights the positive class, γ emphasizes hard examples, and the margin specifies the desired score separation between paired examples. The checkpoint with the best validation model-selection score was retained for each seed.

Test-time adaptation (TTA) was applied after the supervised checkpoint had been trained and before final scoring on each held-out target. For each target and each seed checkpoint, the model was copied and adapted using only the unlabeled molecules from that target; held-out labels were not used. All model weights were frozen except the affine parameters of the LayerNorm modules. The TTA objective combined entropy minimization, an anchor that penalized deviation from the pre-adaptation probabilities, and a prevalence anchor that preserved the mean prediction level of the seed-ensemble teacher. A validation-selected branch also allowed teacher-guided pseudo-ranking, pseudo-labeling of high-ranked and low-ranked molecules, and perturbation consistency. The TTA grid used 5 or 10 adaptation steps, learning rates of $1\times 10^{-4}$ or $5\times 10^{-4}$, batch size 4096, top-ranked pseudo-positive fractions of 0.01–0.10, a pseudo-negative fraction of 0.50, and input perturbation standard deviations of 0.02 or 0.05 where consistency regularization was enabled. The configuration for each fold was selected only on that fold’s validation split by prioritizing validation P20, then NEF1, then BEDROC; when no meaningful validation early-enrichment gain was observed, an entropy-anchor fallback was allowed only if calibration loss improved without a material ranking decrease. The TASC-VS without TTA variant skipped this adaptation step but retained the same trained checkpoints, feature preprocessing, uncertainty estimation, calibration, and final ranking rule.

After TTA, the five seed-specific probabilities were aggregated for each molecule. The mean adapted probability was the central prediction, and the across-seed standard deviation represented predictive uncertainty. Fold-wise temperature scaling was fitted on the corresponding validation split and then applied to validation and held-out test probabilities. When a validation split contained only one class, the temperature was kept at 1.0. For molecule *i*, the final ranking score was $s_{i}={\bar{p}}_{i,cal}-\lambda \sigma_{i}$, where ${\bar{p}}_{i,cal}$ is the calibrated mean probability, $\sigma_{i}$ is the across-seed standard deviation, and λ controls the uncertainty penalty. The primary analysis used λ=0.50. Because this value was not prospectively registered as an optimum, we added a post hoc sensitivity analysis to assess dependence on this choice. The TASC-VS without UQ variant ranked by calibrated probability alone, whereas the TASC-VS without calibration variant ranked by uncalibrated mean probability minus 0.5 times uncertainty. These components follow standard ideas from test-time adaptation, uncertainty quantification, probabilistic calibration, and proper probabilistic scoring [48–53].

The classical baselines were evaluated within the same benchmark and endpoint framework. Vina served as the docking baseline and was run with AutoDock Vina v1.2.7 using exhaustiveness = 16, num_modes = 1, energy_range = 3, and seed = 20260325; the negated docking affinity was used as the ranking score. RTMScore was applied as a pose-based rescoring baseline to the same Vina-generated poses used by Vina and the TASC family. We therefore refer to this comparator as RTMScore-on-Vina-poses. This same-pose design isolates downstream scoring and decision logic from pose-source variation. Vina+RTMScore-on-Vina-poses consensus was formed by averaging target-wise percentile ranks from the two scores. CarsiDock was included as an end-to-end docking baseline on the same target-specific ligand pool [54]. The VSFlow-ECFP4 baseline ranked test molecules by Tanimoto similarity to training positives using an extended-connectivity fingerprint with diameter 4 (ECFP4), providing a ligand-similarity control without protein structure or docking poses.

We also assessed the feasibility of modern comparators requested during review. GNINA was evaluated as GNINA-on-Vina-poses CNNscore on 22,929 matched molecule-target records across all 15 targets. This is a same-pose convolutional-neural-network rescoring analysis, not a full GNINA docking run. The DrugCLIP reconstructed-pocket analysis covered all 23,250 records and is reported as a sensitivity analysis because a matched official end-to-end reproduction was not available [55]. These analyses are reported separately from the main comparator table.

Finally, we added an external Directory of Useful Decoys, Enhanced (DUD-E) stress test [44]. This analysis used eight DUD-E targets for which active and decoy inputs and feature generation were complete (akt1, ampc, cp3a4, cxcr4, gcr, hivpr, hivrt, and kif11), yielding 25,546 molecule-target records. The TASC-VS model trained on the LIT-PCBA folds was applied without retraining, and its results were compared with external Vina and RTMScore-on-Vina-poses scores.

### Primary variables and endpoints

The primary independent variable was method type, defined as the screening package or ranking strategy label. This included the TASC-family variants and the classical baselines. The primary outcome was not broad generalization, mechanistic interpretability, or theoretical calibration optimality, but deployment-oriented top-k screening quality: whether a method can more effectively move experimentally valuable candidates into the highest-priority ranks when follow-up resources are limited.

To reflect this objective, evaluation metrics were organized into three tiers. The first tier comprised the headline metrics precision at the top 20 ranked candidates (P20), false positives among the top 20 ranked candidates (FP20), normalized enrichment factor at 1% (NEF1), and Boltzmann-enhanced discrimination of receiver operating characteristic (BEDROC), which summarize overall screening behavior on the frozen benchmark. The second tier comprised paired target-level deployment metrics, especially per-target differences relative to RTMScore-on-Vina-poses, with emphasis on ΔP20 and ΔFP20, to determine whether any aggregate advantage was also supported at the target level. The third tier comprised fixed-budget decision metrics, including P@20, P@50, P@100, FP@20, FP@50, and FP@100, which quantify the ability of each method to prioritize high-value candidates under different budget limits while tracking the associated false-positive burden. This tiered endpoint structure kept the analysis aligned with the deployment question rather than with an open-ended comparison of theoretical properties.

P@*k* was defined as the proportion of true positives among the top-$k_{eff}$ ranked candidates for a target, and FP@*k* as the number of false positives among those candidates. Here, $k_{eff}=\min(k,n)$, where *n* is the number of available candidates for that target, and $TP_{k_{eff}}$ is the number of true positives among the selected candidates. Thus, targets with fewer than *k* candidates were normalized by the number of available ranked candidates rather than by the nominal budget.


$$\begin{array}{c} P@k=\frac{TP_{k_{eff}}}{k_{eff}} \end{array}$$ (1)

$$\begin{array}{c} FP@k=k_{eff}-TP_{k_{eff}} \end{array}$$ (2)

P20 and FP20 were the most important deployment descriptors because they map most directly to the practical question of whether the top 20 candidates are worth experimental follow-up under highly constrained resources. However, P20 and FP20 are not independent evidence: at a fixed effective cutoff, they are two scales for the same top-*k* hit count, with P20 emphasizing hit yield and FP20 emphasizing wasted validation slots. For this reason, FP20 was used as a descriptive decision-burden scale and was not treated as a separate inferential endpoint from P20. NEF1 and BEDROC were retained as early-enrichment summary metrics [56].

Supplementary analyses also report area under the receiver operating characteristic curve (AUROC), area under the precision-recall curve (AUPRC), expected calibration error (ECE), Brier score, and negative log-likelihood (NLL). AUROC and AUPRC describe global discrimination, whereas ECE, Brier score, and NLL describe probability reliability. These metrics provide supporting context for the fixed-budget endpoints. Reliability profiles across the TASC-family variants are shown in S2 Fig.

### Data processing, quality control, and bias control

Raw data processing followed a fixed fold-level procedure. Within each fold, validation and held-out test data were processed using the same feature definitions, the same imputation rules, and the same scaling parameters. Molecule-level probabilities were then recomputed from multiple saved model states to derive fold-specific mean predicted probabilities and uncertainty estimates. This ensured that uncertainty estimation and calibration remained tied to the same fold structure used for evaluation rather than being imposed retrospectively at the full-cohort level. When a validation split contained only one class, unstable calibration fitting was not attempted and a neutral calibration setting was retained instead.

Under the current comparison scheme, downstream ranking scores were constructed through a fixed sequence: multi-seed probability aggregation, fold-wise calibration, uncertainty estimation, and deterministic within-target ranking. Duplicate target-method-ligand records were explicitly disallowed. Ranking was performed first by descending score and then by a stable secondary tie-breaking rule, typically the ligand identifier, to prevent molecules with identical scores from being randomly shuffled in or out of the top-*k* set.

For metrics that are undefined under sparse target composition, the study used an explicit missingness policy rather than artificial default values. Metrics requiring positive examples were treated as undefined on zero-positive targets, and probability-reliability metrics were likewise treated as undefined when probability outputs were unavailable. To avoid numerical instability, probabilities were clipped to the open interval $\left(10^{-15},1-10^{-15}\right)$ before downstream evaluation. When a target contained fewer molecules than a predefined cutoff *k*, top-*k* evaluation used an effective cutoff defined as follows.


$$\begin{array}{c} k_{eff}=\min(k,n) \end{array}$$ (3)

These rules were adopted to distinguish undefined from zero and to reduce the chance that numerical artifacts would be mistaken for genuine screening effects. They also explain why the manuscript reports both the all-target deployment set and the positive-target subset: the former better reflects deployment burden, whereas the latter better supports stable interpretation of enrichment-type and some probability-dependent metrics.

Quality control and bias control operated at four levels. First, headline results were generated from one reporting scheme for the main text and supplementary tables. Second, the 15-target benchmark subset used fixed target membership and fold structure, preventing post hoc changes to the evaluation universe. Third, TASC-VS full was treated as the predefined full-package reference in the main comparison, with component variants used for ablation-level interpretation. Fourth, duplicate target-method-ligand records and unstable ranking ties were handled deterministically. Together, these rules reduced risks of score-definition drift, leakage through evaluation bookkeeping, and mechanism overinterpretation.

LIT-PCBA has recently been audited for data leakage and molecular redundancy across its published splits, including identical inactive compounds, active-analogue overlap, and low-diversity query sets [57]. The fixed outer folds used here prevent held-out target labels from entering local TASC-layer training. To examine obvious similarity artefacts, we added the VSFlow-ECFP4 ligand-similarity control and the overlap-filtered retained-subset analysis described below. These analyses provide a stricter benchmark-local sensitivity check, although they do not establish performance on novel chemotypes.

To provide a stricter overlap-controlled sensitivity analysis, we filtered the benchmark outputs under two retained-subset definitions: exact-clean and scaffold-clean. This supplementary analysis did not rebuild the benchmark and did not retrain the model; it reevaluated the retained molecules using the same ranking and paired-comparison metrics as the main analysis. The overlap-filtered analysis compared TASC-VS full with RTMScore-on-Vina-poses and Vina. Because retained positive-target coverage was limited, especially for scaffold-clean, the analysis was treated as secondary robustness context rather than as a replacement primary benchmark.

### Statistical analysis

Statistical analysis used target-level paired comparisons in addition to aggregate means. Key comparisons emphasized ΔP20 and the corresponding descriptive ΔFP20 scale relative to RTMScore-on-Vina-poses. This analysis tested whether the aggregate pattern reflected gains across multiple targets or a small number of extreme values.

Practical deployment value was analyzed through fixed-budget top-*k* metrics at *k* = 20, 50, and 100, comparing precision and false-positive burden under realistic screening conditions in which only a limited number of candidates can be experimentally followed up.

Inference used target-level paired statistics. We performed 10,000 target-level bootstrap resamples and report 95% percentile bootstrap confidence intervals. Paired differences were also evaluated with two-sided Wilcoxon signed-rank tests, discarding zero differences under the standard Wilcoxon convention [58,59]. Because multiple metrics and comparators were examined, we applied Holm family-wise error-rate correction and Benjamini–Hochberg false-discovery-rate correction across the independent inferential endpoint family [60,61]. FP@*k* metrics were excluded from this family because they re-express the same top-*k* hit counts as P@*k*; they were retained as descriptive false-positive burdens. Raw Wilcoxon *p* values are reported descriptively, and interpretation is based on effect direction, bootstrap intervals, multiplicity-corrected results, component analyses, sensitivity analyses, and external stress-test behaviour. Detailed corrected results are reported in S2 Table.

Within this framework, TASC-VS full is interpreted as the method with the most favourable calibration-aware top-*k* profile among the evaluated comparators on the curated LIT-PCBA subset.

## Results

### The frozen benchmark provided an auditable and deployment-relevant evaluation setting

We first examined benchmark coverage. The curated 15-target LIT-PCBA subset contains 23,250 evaluated molecule-target records and 181 positives overall. It represents approximately 5.6% of molecules and 2.3% of actives from the published LIT-PCBA benchmark. Inclusion required complete target metadata, fold assignment, labels, Vina poses, RTMScore outputs, and TASC-VS input features. Target-level coverage is reported in S1 Table.

Only seven of the 15 evaluated targets contained at least one positive, whereas eight targets had zero positives. We therefore used an all-target deployment set to assess portfolio-level screening utility and false-positive burden, and a positive-target subset for enrichment endpoints that require positive support. Five fixed outer folds with fixed validation rotation preserved target-level isolation for training, calibration, and held-out testing.

### TASC-VS full showed the most favourable headline profile on the evaluated benchmark

**Fig 2:**
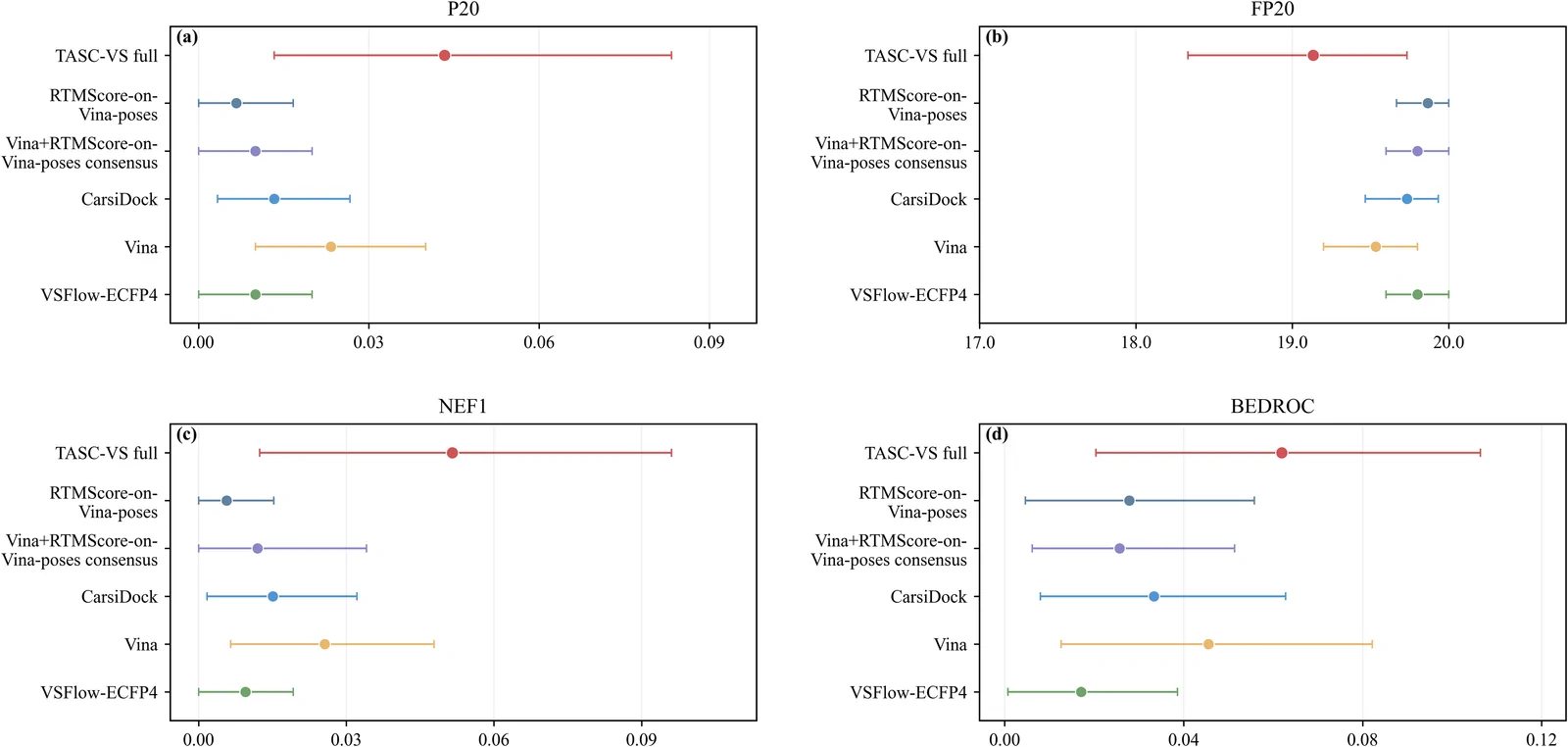
Comparison of TASC-VS full and the main comparators on the four headline benchmark metrics. Points denote across-target means, and horizontal error bars denote 95% target-level bootstrap confidence intervals. P20 and FP20 were bootstrapped over all 15 targets, whereas NEF1 and BEDROC were bootstrapped over the seven targets with positives.

We then evaluated the headline results. As shown in Fig 2 and Table 1, TASC-VS full produced the most favourable headline profile among the evaluated main comparators, although the absolute top-*k* gains were modest. On the all-target deployment set, TASC-VS full achieved P20 = 0.0433 and FP20 = 19.133. Because P20 and FP20 are two scales for the same top-20 hit count, these values indicate a small increase in mean hit yield and the corresponding decrease in false-positive burden. The P20/FP20 values were 0.0067/19.867 for RTMScore-on-Vina-poses, 0.0233/19.533 for Vina, 0.0100/19.800 for Vina+RTMScore-on-Vina-poses consensus, 0.0133/19.733 for CarsiDock, and 0.0100/19.800 for VSFlow-ECFP4.

**Table 1:** Headline benchmark values for TASC-VS full and the main comparators on the curated LIT-PCBA subset.

Metric	N targets	TASC-VS full	RTMScore-on-Vina-poses	Vina	Vina+RTMScore-on-Vina-poses consensus	CarsiDock	VSFlow-ECFP4
NEF1	7	0.0515	0.0057	0.0256	0.0120	0.0151	0.0095
BEDROC	7	0.0619	0.0279	0.0456	0.0257	0.0334	0.0171
P20	15	0.0433	0.0067	0.0233	0.0100	0.0133	0.0100
FP20	15	19.133	19.867	19.533	19.800	19.733	19.800

The enrichment-oriented metrics showed the same direction within the positive-target subset. Because NEF1 and BEDROC require positive support, they were evaluated on the seven positive targets. TASC-VS full had NEF1 = 0.0515 and BEDROC = 0.0619. The corresponding values were lower for RTMScore-on-Vina-poses (0.0057 and 0.0279), Vina (0.0256 and 0.0456), Vina+RTMScore-on-Vina-poses consensus (0.0120 and 0.0257), CarsiDock (0.0151 and 0.0334), and VSFlow-ECFP4 (0.0095 and 0.0171).

Aggregate summaries do not show whether an effect is distributed across targets or driven by a few target-specific gains. We therefore examined paired target-level comparisons.

### Paired target-level analyses argued against an aggregate-illusion explanation

**Fig 3:**
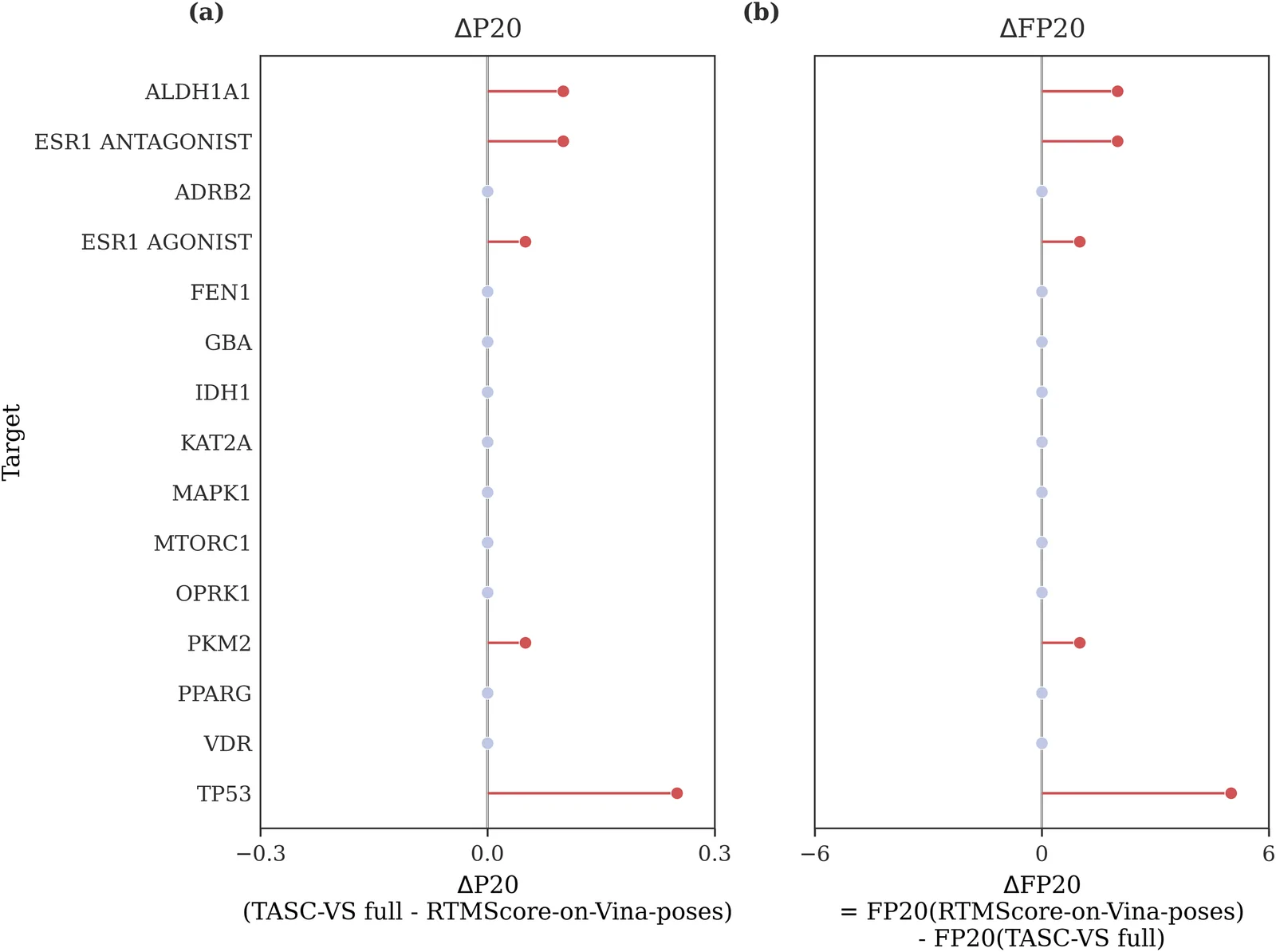
Paired per-target differences between TASC-VS full and RTMScore-on-Vina-poses on P20 and FP20.

We compared TASC-VS full with RTMScore-on-Vina-poses using P20 and the corresponding FP20 burden scale. We defined ΔP20 as P20(TASC-VS full)−P20(RTMScore-on-Vina-poses) and ΔFP20 as FP20(RTMScore-on-Vina-poses)−FP20(TASC-VS full). Positive values therefore indicate a more favourable top-20 profile for TASC-VS full. As shown in Fig 3, five targets showed positive shifts in both measures, while the remaining ten targets were tied or nearly tied. The gain was therefore concentrated in a subset of targets, with broad neutrality elsewhere.

We summarized target-level evidence using win/tie/loss counts, mean benefit, bootstrap confidence intervals, raw Wilcoxon signed-rank tests, and Holm/BH-corrected values for the independent inferential endpoints (Table 2; S2 Table). Against RTMScore-on-Vina-poses, TASC-VS full improved P20 by a mean of 0.0367 with win/tie/loss counts of 5/10/0 and a 95% bootstrap CI of 0.0067–0.0733. The raw Wilcoxon value was *p* = 0.026, whereas the corrected values were Holm *p* = 1.0 and BH *q* = 0.322. The corresponding FP20 benefit was 0.733 fewer false positives among the top 20 on average; this is a descriptive transformation of the same hit-count result. Relative to Vina, Vina+RTMScore-on-Vina-poses consensus, CarsiDock, and VSFlow-ECFP4, P20 benefits were also modest and nonnegative on average. NEF1 and BEDROC favoured TASC-VS full on average against RTMScore-on-Vina-poses, although the BEDROC interval crossed zero.

**Table 2:** Corrected paired target-level statistics for TASC-VS full against the main comparators. Raw Wilcoxon values are descriptive; Holm and BH corrections are shown only for independent inferential endpoints. FP20 is the descriptive false-positive burden for the same top-20 hit count as P20.

Comparison	Metric	N	Win/Tie/Loss	Mean benefit	95% bootstrap CI	Raw Wilcoxon *p*	Holm *p*	BH *q*
TASC-VS full vs RTMScore-on-Vina-poses	NEF1	7	3/4/0	0.0458	0.0106 to 0.0934	0.1250	1.000	0.322
TASC-VS full vs RTMScore-on-Vina-poses	BEDROC	7	5/0/2	0.0340	−0.0225 to 0.0902	0.2969	1.000	0.453
TASC-VS full vs RTMScore-on-Vina-poses	P20	15	5/10/0	0.0367	0.0067 to 0.0733	0.0262	1.000	0.322
TASC-VS full vs RTMScore-on-Vina-poses	FP20 descriptive	15	5/10/0	0.7333	0.1333 to 1.4667	0.0261	–	–
TASC-VS full vs Vina	P20	15	2/13/0	0.0200	0.0000 to 0.0533	0.1575	1.000	0.322
TASC-VS full vs Vina	FP20 descriptive	15	2/13/0	0.4000	0.0000 to 1.0667	0.1575	–	–
TASC-VS full vs Vina+RTMScore-on-Vina-poses consensus	P20	15	5/10/0	0.0333	0.0067 to 0.0700	0.0260	1.000	0.322
TASC-VS full vs Vina+RTMScore-on-Vina-poses consensus	FP20 descriptive	15	5/10/0	0.6667	0.1333 to 1.4000	0.0260	–	–
TASC-VS full vs CarsiDock	P20	15	4/11/0	0.0300	0.0033 to 0.0633	0.0462	1.000	0.322
TASC-VS full vs CarsiDock	FP20 descriptive	15	4/11/0	0.6000	0.0667 to 1.2667	0.0462	–	–
TASC-VS full vs VSFlow-ECFP4	P20	15	5/10/0	0.0333	0.0067 to 0.0700	0.0260	1.000	0.322
TASC-VS full vs VSFlow-ECFP4	FP20 descriptive	15	5/10/0	0.6667	0.1333 to 1.4000	0.0260	–	–

These paired analyses indicate modest gains on a subset of targets and neutrality elsewhere. A broader target-level benefit map is provided in S1 Fig. We next examined fixed-budget decision quality.

### The benchmark advantage translated into better fixed-budget decision quality

**Fig 4:**
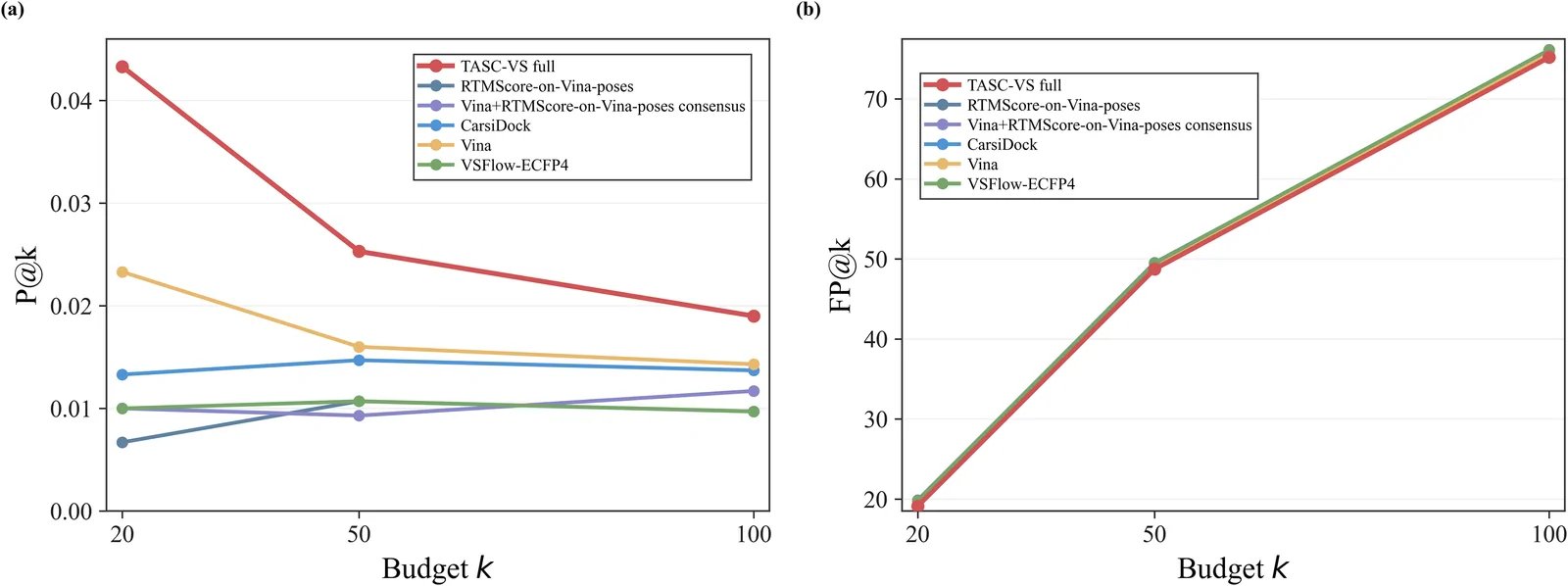
Comparison of fixed-budget decision quality across TASC-VS full and the main comparators at *k* = 20, 50, and 100.

We next examined fixed-budget decisions at *k* = 20, 50, and 100, using P@*k* to quantify hit yield and FP@*k* to express false-positive burden. As shown in Fig 4 and Table 3, TASC-VS full had the most favourable P@*k* profile among the evaluated main comparators across the three budgets. At *k* = 20, TASC-VS full increased mean P@*k* by 0.0200 relative to Vina and by 0.0367 relative to RTMScore-on-Vina-poses.

**Table 3:** Fixed-budget P@*k* and FP@*k* values corresponding to Fig 4.

Metric	N targets	TASC-VS full	RTMScore-on-Vina-poses	Vina	Vina+RTMScore-on-Vina-poses consensus	CarsiDock	VSFlow-ECFP4
P@20	15	0.0433	0.0067	0.0233	0.0100	0.0133	0.0100
P@50	15	0.0253	0.0107	0.0160	0.0093	0.0147	0.0107
P@100	15	0.0190	0.0097	0.0143	0.0117	0.0137	0.0097
FP@20	15	19.133	19.867	19.533	19.800	19.733	19.800
FP@50	15	48.733	49.467	49.200	49.533	49.267	49.467
FP@100	15	75.200	76.133	75.667	75.933	75.733	76.133

As the budget increased from 20 to 100, P@*k* decreased for all methods and FP@*k* increased because deeper portions of each ranked list were included. TASC-VS full preserved the highest P@*k* among the evaluated main comparators at each budget. P@*k* and FP@*k* describe the same selected set on different scales, so these results support the same modest top-*k* pattern.

The fixed-budget analysis shows that the benchmark-level and target-level pattern is retained across multiple experimental budgets on the curated LIT-PCBA subset.

### Component and sensitivity analyses supported a consistent within-benchmark pattern

We next examined the contribution of individual components and the sensitivity of the result to target composition and the uncertainty penalty (Table 4; S3 Table). Calibration reduced ECE from 0.0548 to 0.0271, Brier score from 0.0198 to 0.0167, and NLL from 0.0960 to 0.0867 while leaving P20 unchanged at 0.0400 when uncertainty was not used. Uncertainty penalization increased P20 from 0.0400 to 0.0433 and reduced FP20 from 19.200 to 19.133. TTA produced a smaller reliability change: relative to calibration+UQ without TTA, P20 and FP20 were unchanged and ECE changed from 0.0271 to 0.0269. The reliability profiles are shown in S2 Fig. Thus, calibration mainly improved probability reliability, whereas uncertainty penalization contributed a small top-20 ranking change.

**Table 4:** Component ablation summary for TASC-family variants on the frozen benchmark.

Variant	NEF1	BEDROC	P20	FP20	ECE	Brier	NLL
Base ensemble	0.0572	0.0645	0.0400	19.200	0.0548	0.0198	0.0960
Calibration only (w/o UQ)	0.0572	0.0645	0.0400	19.200	0.0271	0.0167	0.0867
UQ only (w/o calibration)	0.0578	0.0672	0.0433	19.133	0.0548	0.0198	0.0960
Calibration+UQ without TTA	0.0515	0.0619	0.0433	19.133	0.0271	0.0167	0.0867
TASC-VS full (TTA+calibration+UQ)	0.0515	0.0619	0.0433	19.133	0.0269	0.0166	0.0864

The post hoc uncertainty-penalty analysis showed that the P20/FP20 pattern was stable across the evaluated λ values. At λ=0, P20/FP20 were 0.0400/19.200. Across λ=0.25, 0.50, 0.75, and 1.00, P20 remained 0.0433 and FP20 remained 19.133, while NEF1 and BEDROC varied modestly. Thus, the primary setting λ=0.50 is representative of the evaluated uncertainty-penalty range. Leave-one-target-out analysis also showed stable effect direction within this benchmark. Against RTMScore-on-Vina-poses, the mean P20 benefit remained nonnegative in all 15 exclusions, ranging from 0.0214 to 0.0393; NEF1 benefit ranged from 0.0258 to 0.0535, and BEDROC benefit ranged from 0.0134 to 0.0531. Against Vina, P20 benefit also remained nonnegative in all 15 exclusions, with a smaller range of 0.0071–0.0214.

Because only seven targets contained positives, we repeated the paired interpretation on the positive-target subset as an exploratory sensitivity analysis. Against RTMScore-on-Vina-poses, the P20 benefit was 0.0786 with a 95% bootstrap CI of 0.0286–0.1429 and win/tie/loss counts of 5/2/0. The raw Wilcoxon value was *p* = 0.0625, and corrected values were Holm *p* = 1.0 and BH *q* = 0.395. This analysis supports the same effect direction without strengthening the statistical claim.

### Comparator fairness, modern-baseline feasibility, and runtime cost

The comparator analysis clarified three fairness points (S4 Table). First, RTMScore was applied to the same Vina-generated poses used by Vina and TASC-VS, so we report it as RTMScore-on-Vina-poses. Second, the VSFlow-ECFP4 ligand-similarity control remained below TASC-VS full on the evaluated benchmark (NEF1 = 0.0095, BEDROC = 0.0171, P20 = 0.0100, FP20 = 19.800). Third, GNINA-on-Vina-poses CNNscore was completed on 22,929 matched records and gave NEF1 = 0.0240, BEDROC = 0.0458, P20 = 0.0200, and FP20 = 19.600; the corresponding matched TASC-VS full values were NEF1 = 0.0517, BEDROC = 0.0627, P20 = 0.0400, and FP20 = 19.200. The DrugCLIP reconstructed-pocket sensitivity analysis covered all 23,250 records and gave NEF1 = 0.0017, BEDROC = 0.0097, P20 = 0.0067, and FP20 = 19.867. Because this was not an official end-to-end DrugCLIP reproduction, it is reported as a feasibility result.

Runtime was also considered when interpreting deployment feasibility. Downstream TASC-VS calibration+UQ inference and evaluation processed all 23,250 records in 10.1 seconds on a central processing unit (CPU). Serial throughput estimates from stratified samples were approximately 6.6 hours for RTMScore-on-Vina-poses rescoring, 9.5 hours for GNINA-on-Vina-poses CNNscore, and 257 hours for AutoDock Vina pose generation across the 23,250 records. These values depend on hardware and implementation, but they indicate that pose generation and deep rescoring dominate computational cost.

### A sampled DUD-E stress test defined an external generalization boundary

Finally, we evaluated transfer to a sampled DUD-E stress test comprising eight targets (akt1, ampc, cp3a4, cxcr4, gcr, hivpr, hivrt, and kif11) and 25,546 molecule-target records. Vina was stronger than TASC-VS full for early enrichment and fixed-budget prioritization (Table 5; S5 Table). Vina achieved NEF1 = 0.311, BEDROC = 0.302, P20 = 0.419, and FP20 = 11.625. By contrast, TASC-VS full with λ=0.50 achieved NEF1 = 0.075, BEDROC = 0.115, P20 = 0.138, and FP20 = 17.250; the λ=1.00 setting improved these values slightly to NEF1 = 0.083, BEDROC = 0.127, P20 = 0.150, and FP20 = 17.000, but remained below Vina.

**Table 5:** External sampled DUD-E stress-test results. Vina outperformed TASC-VS full on early enrichment and fixed-budget prioritization.

Method	Targets	Molecule-target records	NEF1	BEDROC	P20	FP20
Vina external DUD-E	8	25,546	0.311	0.302	0.419	11.625
RTMScore-on-Vina-poses external DUD-E	8	25,546	0.163	0.201	0.206	15.875
TASC-VS full external DUD-E, λ=0.50	8	25,546	0.075	0.115	0.138	17.250
TASC-VS full external DUD-E, λ=1.00	8	25,546	0.083	0.127	0.150	17.000

The DUD-E result shows that the calibration-aware top-*k* pattern observed on the curated LIT-PCBA subset does not transfer automatically to a different benchmark. The contrast is consistent with benchmark dependence, including differences in target composition and candidate distributions, although the present analysis does not isolate a single cause.

### Overlap filtering provided a benchmark-local robustness check

**Fig 5:**
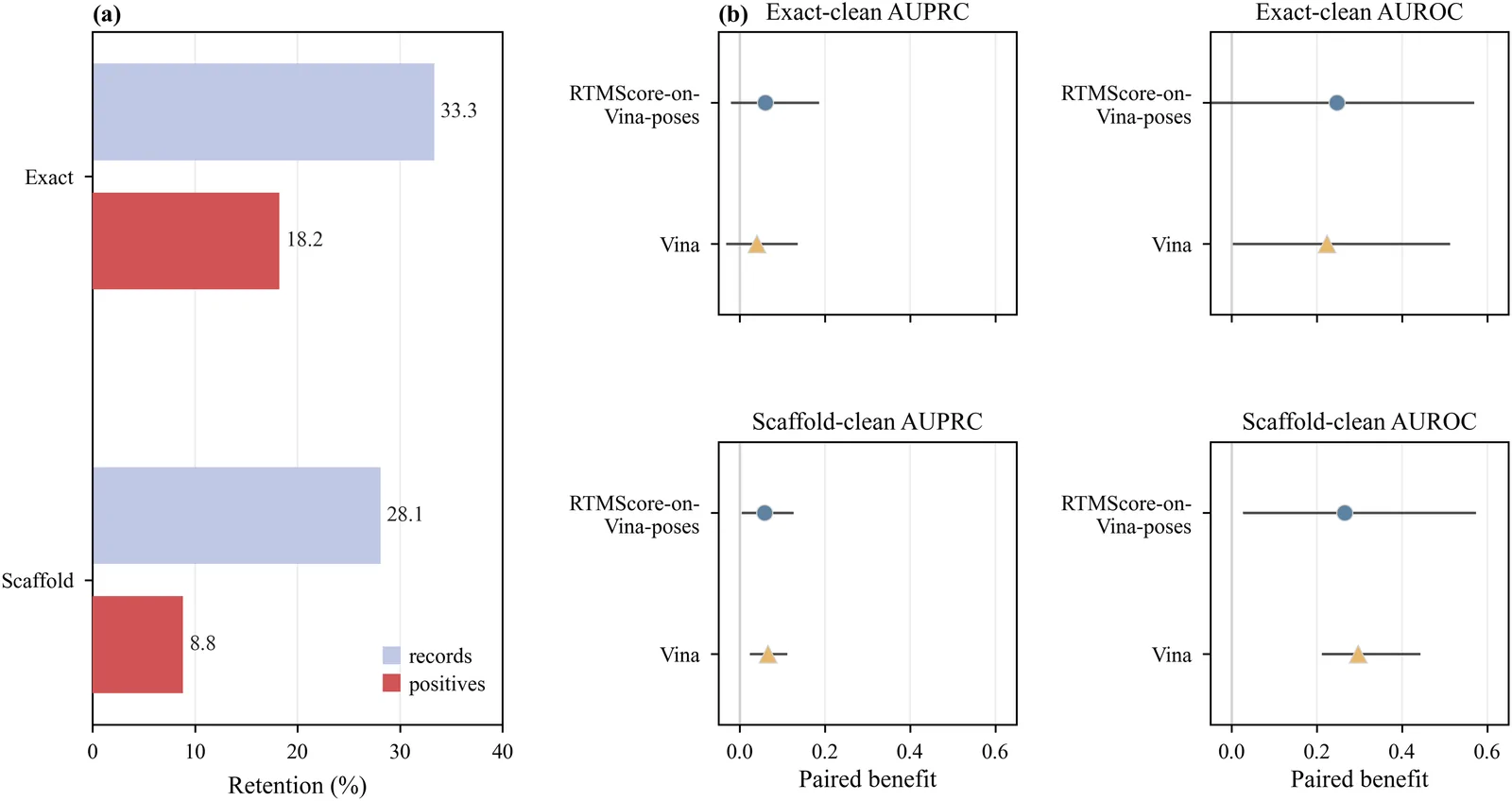
Retained coverage and paired effects for the secondary overlap-filtered analysis under exact-clean and scaffold-clean settings.

To examine stricter overlap filtering, we analysed two retained subsets: exact-clean and scaffold-clean. Fig 5 summarizes retained coverage and paired effects. Both subsets retained 11 targets and four positive targets; exact-clean retained 7,752 records and 33 positives, whereas scaffold-clean retained 6,537 records and 16 positives. Comparator-specific statistics are reported in S3 Table. Given the four positive targets, this analysis provides secondary benchmark-local context.

On the exact-clean subset, the mean AUPRC benefit relative to RTMScore-on-Vina-poses was 0.0596 (95% bootstrap CI, −0.0209 to 0.1860) and the AUROC benefit was 0.2468 (95% bootstrap CI, −0.0501 to 0.5693). On the scaffold-clean subset, the corresponding mean AUPRC and AUROC benefits were 0.0579 (95% bootstrap CI, 0.0045 to 0.1264) and 0.2654 (95% bootstrap CI, 0.0262 to 0.5734). The exact-clean subset also retained a higher TASC-VS P20 than RTMScore-on-Vina-poses and Vina. The scaffold-clean subset retained a higher TASC-VS P20 than Vina but showed more mixed enrichment behaviour. These results provide a benchmark-local sensitivity check against obvious overlap artefacts. A route-level visualization is provided in S3 Fig.

## Discussion

The revised results support a narrower message than the original submission. TASC-VS full showed a modest top-*k* advantage within the curated LIT-PCBA subset, but the evidence is better read as calibration-aware decision support than as a general ranking-superiority claim. This distinction matters because the workflow combines ranking, probability reliability, and uncertainty-aware triage under fixed experimental budgets.

The component analyses suggest that the main value came from reliability and uncertainty handling rather than from TTA alone. Calibration improved ECE, Brier score, and NLL, uncertainty penalization produced a small top-20 change, and TTA had only minor effects on reliability. The λ sensitivity, leave-one-target-out analysis, and positive-target-only analysis support effect direction within this subset without strengthening the statistical claim.

The main limitations are benchmark scope and comparator scope. The curated subset is small relative to full LIT-PCBA and contains positives in only seven targets; RTMScore-on-Vina-poses, GNINA-on-Vina-poses CNNscore, and the DrugCLIP reconstructed-pocket sensitivity analysis were evaluated under constrained settings; and DUD-E favored Vina. These limits define where the current evidence stops.

## Conclusion

In conclusion, TASC-VS full provides calibration-aware fixed-budget top-*k* decision support on the curated LIT-PCBA subset, with modest and directionally consistent benefits rather than multiplicity-corrected statistical superiority. Because Vina outperformed TASC-VS full on the sampled DUD-E stress test, broader deployment should be treated as a separate validation question.

## Supporting information

S1 Fig Broader target-level benefit map across headline metrics and evaluated comparators. This figure extends the primary paired analysis using direct per-target differences for RTMScore-on-Vina-poses, Vina+RTMScore-on-Vina-poses consensus, CarsiDock, Vina, and VSFlow-ECFP4.
(PNG)

S2 Fig Supplementary reliability profiles across TASC-family component variants. This figure compares ECE, Brier score, and NLL across the calibration+UQ, base-ensemble, calibration-only, and UQ-only configurations.
(PNG)

S3 Fig Detailed overlap-filtered analysis. This figure provides a route-level view of pairwise effects for TASC-VS calibration+UQ under the exact-clean and scaffold-clean retained-subset definitions.
(PNG)

S1 Table Benchmark coverage analysis. This table reports the curated LIT-PCBA subset by target, including evaluated molecule-target records, positive counts, positive-target status, and retention relative to the published LIT-PCBA benchmark.
(XLSX)

S2 Table Corrected paired statistical analysis. This table reports target-level paired statistics, raw Wilcoxon values, Holm correction, and Benjamini–Hochberg correction for the independent inferential endpoint family; FP@*k* metrics are retained as descriptive burden scales.
(XLSX)

S3 TableComponent, sensitivity, and retained-subset analyses.This table combines component ablation, TTA sensitivity, λ sensitivity, leave-one-target-out sensitivity, positive-target analysis, and overlap-filtered retained-subset summaries.(XLSX)

S4 Table Comparator fairness, feasibility, and runtime. This table documents the RTMScore-on-Vina-poses same-pose design, VSFlow-ECFP4 baseline, GNINA and DrugCLIP feasibility results, and runtime-cost summaries.
(XLSX)

S5 Table Sampled DUD-E external stress test. This table reports eight-target DUD-E stress-test coverage, method-level metrics, per-target metrics, and feature-generation summary.
(XLSX)
